# The Genetics of Amyloid Deposition: A Systematic Review of Genome-Wide Association Studies Using Amyloid PET Imaging in Alzheimer’s Disease

**DOI:** 10.3390/jimaging11080280

**Published:** 2025-08-19

**Authors:** Amir A. Amanullah, Melika Mirbod, Aarti Pandey, Shashi B. Singh, Om H. Gandhi, Cyrus Ayubcha

**Affiliations:** 1 Jefferson Einstein Montgomery Hospital, Norristown, PA 19403, USA; aaa166@jefferson.edu; 2 Department of Radiology, Perelman School of Medicine, University of Pennsylvania, Philadelphia, PA 19104, USA; melmirbod@gmail.com; 3 Department of Medicine, College of Medical Sciences, Bharatpur-10, Chitwan 44200, Nepal; aartipandey516@gmail.com; 4 Department of Radiology, Stanford University School of Medicine, Stanford, CA 94305, USA; drmrshashi@gmail.com; 5 Department of Radiology, Hospital of the University of Pennsylvania, Philadelphia, PA 19104, USA; omgandhi@seas.upenn.edu; 6 Harvard Medical School, Boston, MA 02115, USA; 7 Department of Epidemiology, Harvard T.H. Chan School of Public Health, Boston, MA 02115, USA

**Keywords:** Alzheimer’s disease (AD), genome-wide association study (GWAS), positron emission tomography (PET), amyloid, genetics

## Abstract

Positron emission tomography (PET) has become a powerful tool in Alzheimer’s disease (AD) research by enabling in vivo visualization of pathological biomarkers. Recent efforts have aimed to integrate PET-derived imaging phenotypes with genome-wide association studies (GWASs) to better elucidate the genetic architecture underlying AD. This systematic review examines studies that leverage PET imaging in the context of GWASs (PET-GWASs) to identify genetic variants associated with disease risk, progression, and brain region-specific pathology. A comprehensive search of PubMed and Embase databases was performed on 18 February 2025, yielding 210 articles, of which 10 met pre-defined inclusion criteria and were included in the final synthesis. Studies were eligible if they included AD populations, employed PET imaging alongside GWASs, and reported original full-text findings in English. No formal protocol was registered, and the risk of bias was not independently assessed. The included studies consistently identified *APOE* as the strongest genetic determinant of amyloid burden, while revealing additional significant loci including ABCA7 (involved in lipid metabolism and amyloid clearance), *FERMT2* (cell adhesion), *CR1* (immune response), TOMM40 (mitochondrial function), and *FGL2* (protective against amyloid deposition in Korean populations). The included studies suggest that PET-GWAS approaches can uncover genetic loci involved in processes such as lipid metabolism, immune response, and synaptic regulation. Despite limitations including modest cohort sizes and methodological variability, this integrated approach offers valuable insight into the biological pathways driving AD pathology. Expanding PET-genomic datasets, improving study power, and applying advanced computational tools may further clarify genetic mechanisms and contribute to precision medicine efforts in AD.

## 1. Introduction

Alzheimer’s disease (AD) is a multifactorial neurodegenerative disorder marked pathologically by the buildup of misfolded β-amyloid (Aβ) peptides, neurofibrillary tangles from hyperphosphorylated tau (p-tau), and progressive synaptic loss [1,2,3]. Clinically, AD presents as a slow cognitive deterioration, affecting memory, executive function, language, and visuospatial skills. Diagnosis relies on comprehensive clinical evaluation, neuropsychological testing, and corroborative biomarker evidence, including cerebrospinal fluid assays and advanced neuroimaging such as MRI and PET to detect amyloid pathology. Early identification of subtle cognitive deficits, combined with biomarker profiles, enhances diagnostic accuracy and facilitates timely intervention.

Impaired glucose metabolism is a hallmark of AD pathogenesis, with decreased cerebral glucose uptake, particularly in cerebral regions like the posterior cingulate cortex, which is associated with memory deficits [4]. Genetic factors affecting oxidative stress response and glucose regulation may exacerbate this metabolic dysfunction [5,6,7]. Amyloid-beta accumulation can precede clinical symptoms by decades, but the genetic and metabolic mechanisms underlying this remain incompletely understood [8,9,10]. Advances in neuroimaging, especially amyloid PET, allow in vivo visualization of amyloid plaques and neurodegeneration, correlating strongly with pathological and clinical progression [11,12,13]. Similar genome-wide association approaches have been increasingly applied to tau PET imaging, which detects neurofibrillary tangles more closely linked to cognitive decline. Early tau PET–GWASs have identified both overlapping and distinct genetic variants compared to amyloid PET, highlighting the importance of integrated analyses to better understand Alzheimer’s disease pathogenesis and develop targeted interventions. Although tau PET–GWAS is an emerging and valuable field of study, this review is confined to examining genetic research based on amyloid PET imaging to better understand Alzheimer’s disease mechanisms.

AD heritability is estimated at 58–79% [14], motivating large-scale genetic studies to identify pathogenic mechanisms and biomarkers. The *APOE* ε4 allele, which is present in about 16% of the population, carries the highest genetic risk factor for late-onset Alzheimer’s disease [15]. Environmental and metabolic factors, like cardiovascular disease, diabetes, and traumatic brain injury, also increase risk for late-onset AD [2,16,17]. More than 70 genetic loci linked to Alzheimer’s disease risk have been uncovered through genome-wide association studies (GWASs). Some of these loci have been associated with specific single-nucleotide polymorphisms (SNPs) that correlate with key cerebrospinal fluid biomarkers such as beta-amyloid and phosphorylated tau [18,19,20]. However, known variants, including *APOE* ε4, explain only ~30% of genetic susceptibility, and recent GWASs incorporating amyloid PET imaging have revealed additional genetic loci associated with neuronal metabolism and neurodegeneration, sometimes independently of established clinical risk genes [21,22,23,24]. These results emphasize the value of genetic profiling for risk stratification and the pursuit of targeted, pathology-specific therapies. The purpose of this systematic review is to highlight GWAS investigations using amyloid imaging in AD, focusing on the genes modulating the pathological processes and regional brain involvement.

## 2. Materials and Methods

This systematic review was conducted in accordance with the PRISMA 2020 guidelines. A PICO-based framework was used to define the search strategy and eligibility criteria. No protocol was registered or published prior to conducting this review.

A structured, systematic literature search of PubMed and Embase databases was performed to identify articles combining genome-wide association studies (GWASs) with molecular neuroimaging techniques. The full PICO-structured query is reported in the Supplementary Materials, Table S1. The PubMed search employed the following strategy: ((“Alzheimer Disease” [Mesh] OR “alzheimer” [tiab]) AND (“Positron-Emission Tomography” [Mesh] OR “Positron Emission Tomography” [tiab] OR “PET Imaging” [tiab] OR “PET Scan” [tiab] OR “PET Scans” [tiab])) AND (“Genome-Wide Association Study” [Mesh] OR “Genome Wide Association Study” [tiab] OR “Whole Genome Association Study” [tiab] OR “GWA Study” [tiab] OR “GWA Studies” [tiab] OR GWAS [tiab]), yielding 66 articles. A complementary Embase search using the terms (“genome-wide association study”/exp OR “genome-wide association study”) AND (“alzheimer disease”/exp OR “alzheimer disease”) AND (“positron emission tomography”/exp OR “positron emission tomography”) identified 144 articles on 18 February 2025.

**Figure 1:**
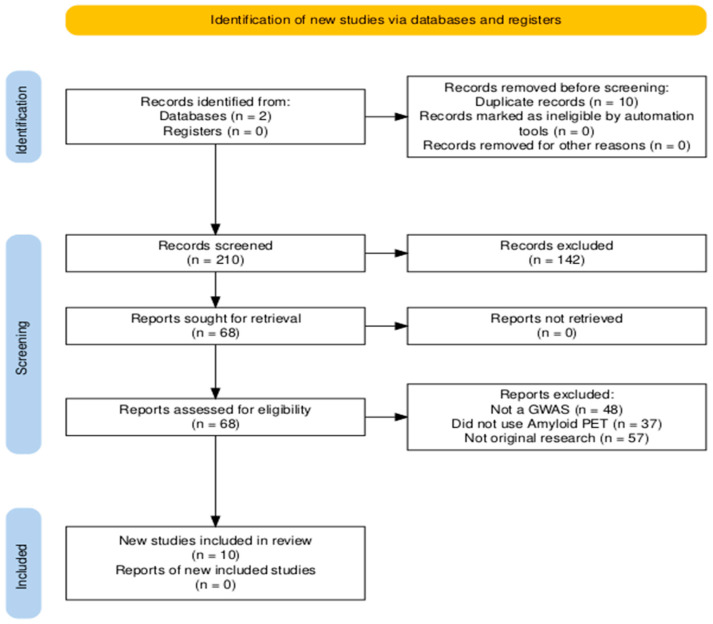
Article processing PRISMA flow diagram outlining the study selection process for the systematic review. A structured search of PubMed and Embase was performed using PICO-based criteria to identify studies that integrated genome-wide association analyses with PET imaging in Alzheimer’s disease. From an initial pool of 210 records (66 from PubMed and 144 from Embase), 68 articles were retained after title and abstract screening. After applying detailed inclusion and exclusion criteria, 10 studies utilizing amyloid PET imaging met all eligibility requirements for full-text review and analysis.

After an initial screening of titles and abstracts by two independent reviewers, 68 relevant articles were selected from the combined total of 210. Strict inclusion criteria were then applied to ensure relevance and quality. Eligible studies were required to (1) include populations diagnosed with AD or at risk for AD, (2) investigate genetic variation associated with AD through GWAS, and (3) utilize amyloid PET imaging techniques to complement genetic data. Studies were excluded if they focused on PET imaging modalities other than amyloid PET (such as FDG PET) without concurrently using amyloid PET-derived imaging phenotypes for genetic association analysis. Studies were required to include AD cases (or individuals with a family history of AD) and controls. The primary goal of the studies must have been to identify genetic variants that influence AD by assessing PET imaging phenotypes. Only original, full-text research articles published in English were included; abstracts, reviews, and non-English papers were excluded (n = 142). No minimum sample size was specified. All included articles met the pre-defined PICO criteria. After removing duplicates, 10 unique articles remained that satisfied all inclusion criteria and were chosen for full-text review (Figure 1).

## 3. Results and Discussion

The integration of amyloid PET imaging with GWASs has enabled the identification of genetic variants influencing AD pathophysiology at a molecular level. Amyloid PET ligands such as [^11^C]PiB and [^18^F]Florbetapir quantify in vivo cerebral β-amyloid deposition, providing a neurobiological endophenotype for genetic association. Across multiple large-scale cohorts, including the Alzheimer’s Disease Neuroimaging Initiative (ADNI), as well as other multiethnic consortia [25], GWASs have consistently shown the *APOE* locus as the major genetic determinant of amyloid burden. However, recent studies have expanded this landscape by uncovering additional genetic loci, such as *ABCA7* and *FERMT2* [25,26], with potential modulatory effects on amyloid accumulation and metabolism. These loci demonstrate different associations across clinical stages, populations, and sex-stratified analyses, underscoring the complex and heterogeneous genetic architecture of amyloid pathology as summarized in Table 1.

## 4. Apolipoprotein-Mediated Lipid Transport

Across studies in the literature, *APOE* remains the strongest and most consistent genetic predictor of amyloid accumulation [27]. In one of the largest genome-wide association studies (GWASs) using amyloid-PET data, Ali and colleagues analyzed 13,409 participants from multiethnic, multicenter cohorts—including the Alzheimer’s Disease Neuroimaging Initiative (ADNI), Memory and Aging Project (MAP), Dominantly Inherited Alzheimer Network (DIAN), and Knight Alzheimer Disease Research Center (Knight ADRC)—and identified several genetic loci significantly associated with cerebral amyloid accumulation and Alzheimer’s disease [27]. As expected, a robust signal was detected at the *APOE* locus on chromosome 19q13.32, with the *APOE* ε4 allele (rs429358) showing the strongest association with amyloid burden (β = 0.35, *p* = 6.2 × 10^−31^). In addition to *APOE* ε4, five novel variants within the *APOE* region—ε2 (rs7412), rs73052335, rs5117, rs1081105, and rs438811—demonstrated significant, independent associations with amyloid deposition. Importantly, race-stratified analyses revealed that the effects of *APOE* ε4 and ε2 were strongest in non-Hispanic whites and weakest among Asian populations, emphasizing the role of ancestry in genetic risk [27].

In a separate study, Yan and colleagues assessed cerebral amyloid buildup in 1000 individuals from three Alzheimer’s research centers and the ADNI cohort using PET imaging with ^11^C-labeled Pittsburgh Compound-B (PiB) [28]. The most significant findings were observed in *APOE*, with *APOE* ε4 being the most strongly associated allele (*p*_meta = 9.09 × 10^−30^; β = 0.18). Together, the leading 15 non-*APOE* SNPs identified by Yan et al., along with *APOE* ε4, accounted for 25 to 35 percent of the variance in amyloid burden across different datasets, with *APOE* ε4 alone accounting for 14 to 17 percent of the variance [28]. These results draw attention to the role of both *APOE* and non-*APOE* genetic variants in regulating amyloid buildup in the brain, and point to the existence of additional ethnicity-based genetic influences that play a role in the development of amyloid accumulation in AD populations.

These findings for *APOE* were highly consistent and reproducible across both multiethnic and single-ancestry cohorts, with effect sizes remaining largely unchanged after adjusting for demographic and clinical covariates. Any variations in allele effect magnitude between ancestry may stem from biological differences and the underrepresentation of non-European populations in most cohorts.

## 5. Mitochondrial Function

The *TOMM40* gene encodes a mitochondrial translocase essential for protein import into mitochondria. *TOMM40* has been linked to the development of Alzheimer’s disease pathogenesis due to its connection with mitochondrial dysfunction. This mitochondrial dysfunction is a key characteristic in neurodegenerative processes contributing to amyloid accumulation and neuronal injury. In Squillario et al.’s comprehensive genetic analysis using data from both ADNI-1 and ADNI-2 cohorts, two genes, *TOMM40* and *GRM7*, emerged as potential contributors to Alzheimer’s disease susceptibility, with *TOMM40* showing the most consistent association across datasets and analytical approaches [29]. Located at the 19q13.32 locus, adjacent to the *APOE* gene, *TOMM40* encodes a mitochondrial membrane translocase essential for importing protein precursors into mitochondria, a process implicated in neurodegenerative pathophysiology [30,31,32,33]. *TOMM40* and *APOE* lie in close proximity on chromosome 19 and show strong linkage disequilibrium in many populations, particularly among individuals of European ancestry, where certain *TOMM40* variants occur in nearly all *APOE* ε4 carriers. This LD is weaker in non-European groups, with distinct haplotype patterns observed across ancestries [34,35]. Single-nucleotide polymorphisms (SNPs) rs2075650 and rs8106922 within *TOMM40* showed significant associations with AD-related traits among individuals carrying the *APOE* ε4 allele. Notably, rs2075650, located within an intron of *TOMM40*, demonstrated significance across both case-control and *APOE* ε4-stratified analyses in ADNI-1.

While *TOMM40* has been linked to amyloid deposition in larger GWASs, its strong linkage disequilibrium with *APOE* makes interpretation challenging. Analyses stratified by *APOE* suggest potential independent effects, but further replication in ancestrally diverse and *APOE*-matched cohorts is needed to confirm these signals.

## 6. Lipid Metabolism and Transport

The *ABCA7* gene is responsible for encoding an ATP-binding cassette transporter involved in lipid homeostasis and phagocytic clearance of amyloid-β [36]. *ABCA7* contributes to Alzheimer’s disease pathogenesis by modulating amyloid deposition and promoting microglial-mediated clearance of pathological aggregates. Apostolova et al. performed a genome-wide association study using data from 977 participants (mean age 74.0 ± 7.5 years, with 54.8% of the participants being male) across the ADNI-1, ADNI-2, and ADNI Grand Opportunity (GO) cohorts, utilizing ^11^C-PiB and [^18^F]Florbetapir PET imaging [25]. The authors identified several genetic loci associated with amyloid deposition. In addition to the well-known impact of the *APOE* ε4 allele, *ABCA7* was independently and strongly linked to amyloid buildup in the brain (χ^2^ = 8.4, FDR-corrected *p* < 0.001). Notably, this association was seen during both asymptomatic and early symptomatic stages of AD, suggesting that *ABCA7* plays a role in the early acceleration of amyloid pathology.

Zhao et al. evaluated the association between 15 variants within the *ABCA7* locus and AD biomarkers using data from the ADNI 2 cohort [26]. While a GWAS previously reported SNPs at this locus, specifically rs3764650 and rs78117248, they were not significantly associated with cerebrospinal fluid (CSF) biomarkers or neuroimaging measures; however, three alternative *ABCA7* variants, rs3752242, rs3752240, and rs4147912, were significantly linked to levels of amyloid accumulation measured by AV-45 ([^18^F]Florbetapir) PET imaging. Further haplotype and subgroup analyses supported these associations, providing additional evidence for the role of these variants in amyloid deposition. Importantly, no significant relationships were found between the *ABCA7* variants and markers of neurodegeneration, such as elevated CSF tau levels, structural brain atrophy, or cerebral hypometabolism as assessed by imaging. This suggests that the *ABCA7* locus contributes to AD risk predominantly through mechanisms related to amyloid-β accumulation, rather than through tau-mediated neuronal injury or broader neurodegenerative processes. This study emphasizes the functional specificity of *ABCA7* in amyloid pathology and stresses the importance of integrating genetic data with imaging phenotypes to elucidate disease mechanisms in AD.

Across two ADNI-based studies, *ABCA7* variants were linked to increased amyloid burden on PET imaging, with effects evident even in the stages of disease. While specific SNP associations differed between the analyses, the results in both studies support a role for *ABCA7* in amyloid accumulation rather than tau-related neurodegeneration, highlighting its functional specificity in AD pathology.

## 7. Peptide Hormone and Transporter Interactions

The *IAPP* gene, also known as islet amyloid polypeptide, encodes amyloidogenic peptide amylin, and the *SLCO1A2* gene, which is responsible for transporting organic anions across the blood–brain barrier, has been found to play a role in AD pathogenesis. Both genes have been linked to Alzheimer’s disease for their potential roles in affecting amyloid-β toxicity and altering brain structure and cognitive function. Roostaei and colleagues carried out a GWAS using data from 678 individuals enrolled in the ADNI-GO cohort to explore genetic factors that influence the link between cortical amyloid levels. This was quantified using [^18^F]Florbetapir PET and cognitive outcomes assessed by the ADAS-Cog [36]. An interaction at rs73069071 within the *IAPP-SLCO1A2* region approached genome-wide significance, with a *p*-value of 6.2 × 10^−8^. This result was replicated in an independent ADNI-1 cohort using ^11^C-PiB PET (one-tailed *p* = 0.028), and a meta-analysis across studies yielded genome-wide significance (*p* = 1.1 × 10^−8^).

Voxel-wise analysis showed that rs73069071 significantly modified the effect of amyloid on temporal lobe cortical thickness (with a family-wise error-adjusted *p* = 0.013). Comparable interactions were also noted over time for ADAS Cog scores, cortical thickness in the left middle temporal region, and the volume of the amygdala. Postmortem data from the Religious Orders Study and Memory and Aging Project further supported the interaction between this SNP and amyloid in relation to global cognitive function (*p* = 0.005, one-tailed). These findings suggest that rs73069071 may influence how amyloid affects cognitive decline, pointing to a possible regulatory role of amylin in AD pathology.

Findings linking the *IAPP* and *SLCO1A2* genes largely stem from analyses by a single group within ADNI, and are supported by replication in a separate cohort. Agreement across different imaging methods and cognitive outcomes supports a biologically plausible effect, but the absence of replication in non-ADNI populations limits broader applicability. These observations raise the possibility that amylin may play a major role in modulating the cognitive effects of amyloid, reinforcing the need for confirmation in larger and more diverse cohorts.

## 8. Lipid-Related Genes from Whole Genome Sequencing

Patel and colleagues conducted whole genome sequencing on a group of 1888 participants of European ancestry, each of whom underwent [^18^F]Florbetapir amyloid PET imaging [37]. Using SKAT-O for gene-based analysis with coding variants as predictors and PET imaging measures as quantitative endophenotypes, the study revealed a genome-wide significant association with *APOE* (*p* = 2.45 × 10^−10^), confirming its well-established role in amyloid deposition. In addition, a set of 26 genes emerged as potential contributors to Alzheimer’s pathology, showing suggestive statistical signals (*p* < 5.0 × 10^−3^); among them were *SCN7A, SH3GL1*, and *MFSD12*. Further functional enrichment pointed toward lipid-binding mechanisms as being closely tied to Aβ deposition, implicating several genes, including *APOE, PITPNM3, AP2A2, and SH3GL1*, with varying degrees of statistical support. These findings suggest a notable connection between lipid metabolism and the accumulation of Aβ, and offer new avenues for promising therapeutic interventions targeting metabolic pathways to mitigate amyloid accumulation and potentially delay or prevent the onset of AD.

## 9. Cell Adhesion

In addition to *APOE* and *ABCA7*, Ali et al. identified *FERMT2* (rs117834516, *p* = 1.1 × 10^−9^) as a locus showing genome-wide significance and colocalization with AD risk [27]. *FERMT2* encodes kindlin-2, a protein involved in integrin-mediated cell adhesion and signaling. Apostolova et al. found a stage-dependent relationship between *FERMT2* and amyloid deposition, with a significant gene-by-diagnosis interaction (χ^2^ = 3.53, FDR-corrected *p* = 0.05). This association was evident in those with mild cognitive impairment, indicating that *FERMT2* may influence amyloid burden in a time-dependent manner during disease progression [25].

## 10. Endocytosis and Vesicle Trafficking

The *SH3GL1* gene codes for a protein that supports clathrin-based endocytosis and intracellular vesicle movement. It has been linked to Alzheimer’s disease through a proposed role in regulating amyloid precursor protein (APP) internalization and processing, thereby affecting amyloid-β generation and accumulation. In the whole genome sequencing study by Patel et al., *SH3GL1* (*p* = 7.56 × 10^−4^) emerged as one of 26 candidate genes with suggestive associations with amyloid burden, but this did not meet the stricter genome-wide significance threshold (*p* < 5 × 10^−8^) applied in GWASs. [37]. While supported by experimental evidence for APP internalization, this genetic association remains preliminary and requires validation in future larger, independent cohorts.

## 11. Immune Response and Inflammation

The *CR1* (complement receptor 1) gene encodes a receptor that clears cellular debris and complement-tagged immune complexes, playing a critical role in modulating innate immune responses. *CR1* has been associated with AD due to its contribution to impaired amyloid-β clearance and chronic neuroinflammation, which may promote plaque accumulation. *CR1* (rs6656401, *p* = 2.4 × 10^−10^) was identified as a genome-wide significant locus by Ali et al. [27]. These PET-based findings align with the broader concept that impairments in the immune system regulation can hinder amyloid-β removal, which in turn fosters amyloid-β accumulation and worsens Alzheimer’s disease pathology.

The *FGL2* gene encodes a fibrinogen-like protein involved in immune regulation and prothrombinase activity, and has likewise been linked to AD pathogenesis through its role in modulating neuroinflammation and amyloid-β clearance, suggesting a protective effect against Aβ deposition. Kim HR et al. identified *FGL2* as a novel locus associated with a reduced risk of Aβ deposition in an ethnically Korean population [38]. Two SNPs on chromosome 7, rs73375428 and rs2903923, reached genome-wide significance and were associated with lower rates of Aβ positivity. Analysis of cis expression quantitative trait locus (eQTL) showed that the minor allele of rs73375428 was linked to reduced *FGL2* expression in the brain, pointing to a possible functional effect on Aβ accumulation.

**Figure 2:**
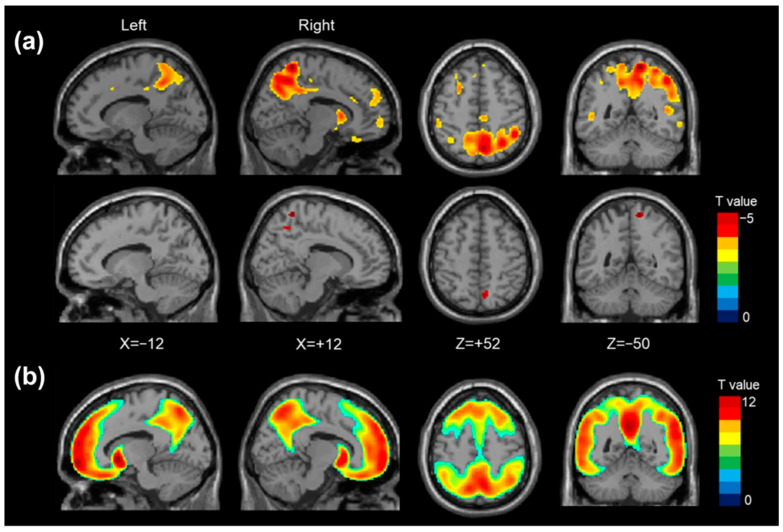
Voxel-wise PET analysis revealed that individuals carrying the minor allele of rs73375428 exhibited reduced amyloid-β deposition (top row: uncorrected *p* < 0.001 with cluster size > 20; bottom row: family-wise error-corrected *p* < 0.05), while those with the *APOE* ε4 allele showed increased Aβ accumulation (family-wise error-corrected *p* < 0.05). Image adapted without modification from Kim et al. [38] under the Creative Commons Attribution 4.0 International License (CC BY). http://creativecommons.org/licenses/by/4.0/ (accessed on 12 June 2025).

This calls attention to *FGL2* as a novel genetic factor associated with reduced risk of Aβ deposition and stresses the importance of including diverse ancestral groups in genetic studies to detect unique ancestry-specific risk and protective factors. The *FGL2* locus may serve as a therapeutic target in efforts to mitigate amyloid pathology in Alzheimer’s disease [38]. As shown in Figure 2, voxel-wise PET analyses revealed significantly decreased amyloid deposition in individuals who are carriers of the minor allele of rs73375428, further supporting the role of *FGL2* as a protective factor against amyloid accumulation in the brain [38].

**Figure 3:**
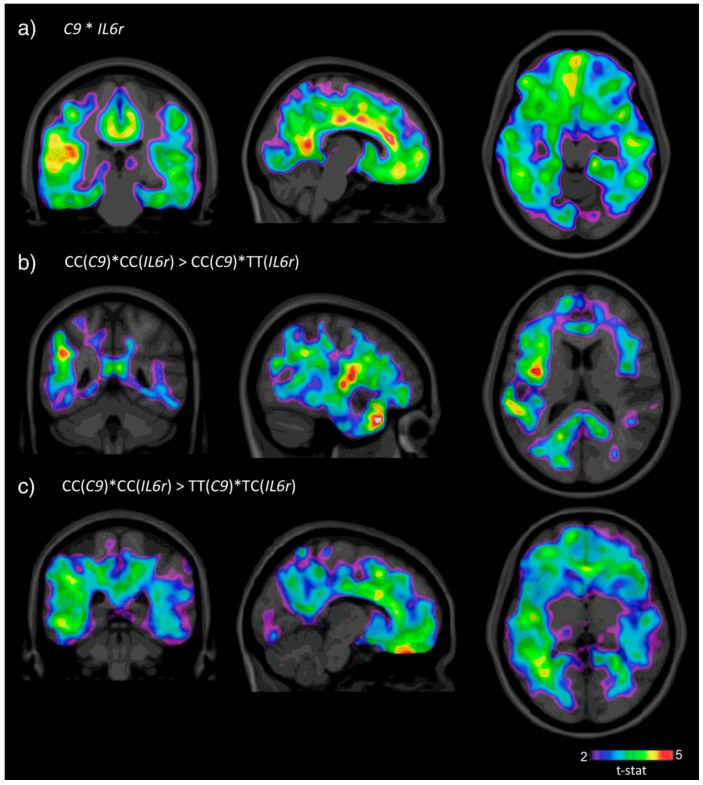
T-statistical maps illustrating genotype-based differences in brain regions with elevated SUVR in individuals carrying the SNP interaction between rs261752 (*C9*) and rs7514452 (*IL6R*). (**a**) SPM overlaid on average structural MRI highlights areas vulnerable to Alzheimer’s disease (AD) pathophysiology. (**b**) Carriers of the CC genotype at both loci (*C9*, *IL6R*) demonstrate increased [18F]florbetapir SUVR in the frontal, parietal, and temporal lobes. (**c**) These same carriers also show greater tracer uptake in areas typically involved in amyloid deposition in AD. All analyses were controlled for sex, diagnostic group, and *APOE* ε4 status, using a statistical threshold of T ≥ 3.2 (*p* ≤ 0.05). Image reprinted without changes from Benedet et al. [40] under the Creative Commons Attribution 4.0 International License (CC BY). http://creativecommons.org/licenses/by/4.0/ (accessed on 12 June 2025).

Aside from single-gene associations, interactions between genes, such as *C9* and *IL6R*, have been shown to modulate amyloid deposition patterns, highlighting the role of immune pathways in amyloid pathology. In a separate investigation, Benedet and colleagues showed that individuals with the CC genotype at both the *C9* and *IL6R* loci exhibited significantly elevated [^18^F]Florbetapir SUVR (standardized uptake value ratio) in regions susceptible to AD, including the frontal, parietal, and temporal cortices [39]. This finding reinforces the importance of SNP–SNP interactions in modulating amyloid deposition and demonstrates how imaging-based genetic approaches can reveal biologically meaningful effects beyond single-gene associations. Figure 3 further illustrates the relevance of gene–gene interactions in AD [40].

## 12. Synaptic Signaling, Cognitive Resilience Loci, and Sex-Specific Genetic Effects

Squillario et al. identified *GRM7* (glutamate metabotropic receptor 7) as one novel gene potentially involved in AD pathogenesis in their *APOE* ε4-stratified analysis of ADNI-1 [29]. *GRM7* plays a key role in presynaptic neurotransmitter modulation and has been implicated in other neuropsychiatric disorders. SNP rs9311976 was associated with AD in ADNI-1, while rs266410 showed significance in ADNI-2, suggesting that synaptic transmission and glutamatergic signaling may contribute to amyloid pathology.

A genome-wide analysis by Eissman et al. identified sex-specific genetic variants that influence resilience to amyloid-related cognitive decline, with the strongest signal located on chromosome 8 [39]. Resilience was defined as better-than-expected cognitive performance for a given amyloid burden. An intronic variant on chromosome 10 interacts with regulatory elements of *GATA3*, *KIN*, and *TAF3*, potentially affecting resilience through astrocyte, oligodendrocyte, and neuronal expression networks. Additional suggestive variants were also found in *CDH18* (in females) and in *MS4A6A, PTK2B, SORL1, KAT8*, and *PICALM* (in males), enriched in chromatin remodeling and RNA processing pathways. These results point to a distinct, sex-specific genetic architecture of resilience in AD.

**Figure 4:**
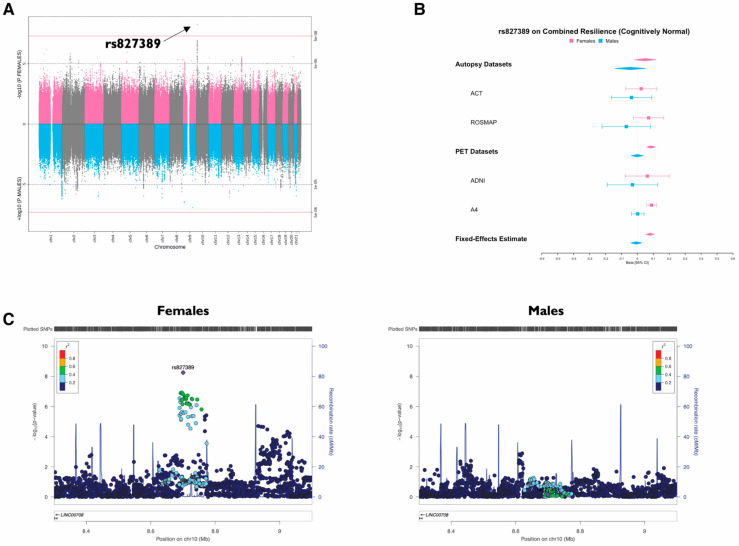
In cognitively intact females, the minor allele of the sex-specific variant rs827389 on chromosome 10 is associated with increased cognitive resilience. (**A**) Miami plot showing female-specific variants in pink and male-specific variants in blue. (**B**) Forest plot illustrating rs827389 associations by sex and cohort, alongside meta-analysis estimates. (**C**) Sex-stratified LocusZoom plots for the chromosome 10 region. Image reprinted without changes from Eissman et al. [39] under the Creative Commons Attribution 4.0 International License (CC BY). http://creativecommons.org/licenses/by/4.0/ (accessed on 12 June 2025).

Beyond GWAS-identified resilience variants, prior imaging genetics studies have shown that *APOE*ε4 carriers with protective genetic backgrounds, such as the *GAB2* haplotype, exhibit higher cerebral metabolism in regions typically vulnerable to AD. Figure 4 illustrates these sex-specific associations, showing that the minor allele of rs827389 on chromosome 10 is significantly linked to higher resilience scores in cognitively normal females, as demonstrated across multiple cohorts using Miami, forest, and locus zoom plots [39].

Sex-specific and resilience-related genetic variants have potential applications in precision medicine by aiding the identification of subgroups with distinct patterns of amyloid accumulation and cognitive changes. These insights could refine clinical trial enrollment criteria and strategies and tailor clinical development of sex-specific therapies. However, presently, these associations are preliminary and require confirmation in larger, more ancestrally diverse cohorts, alongside functional studies to clarify the underlying biologic mechanisms. In summary, these findings indicate that synaptic signaling genes, resilience-associated loci, and sex-specific variants as potentially interrelated factors in shaping amyloid-related cognitive outcomes, but validation in broader and more diverse cohorts remains essential before clinical application.

## 13. Population- and Ancestry-Specific Findings

In a Korean cohort comprising 759 participants, Kim BH et al. conducted a gene-based association study to explore genetic links to Alzheimer’s-related traits. Significant associations with amyloid SUVRs were observed for six genes: *SCRN2, LCMT1, LRRC46*, *MRPL10, OSBPL7*, and *SP6* [38]. The top SNPs included rs4787307 (*LCMT1*), rs9903904(*OSBPL7*), and rs11079797(*SCRN2*). These variants were associated with hippocampal atrophy and cognitive deficits, and pathway analysis identified enrichment in axonal and chemokine signaling networks.

**Figure 5:**
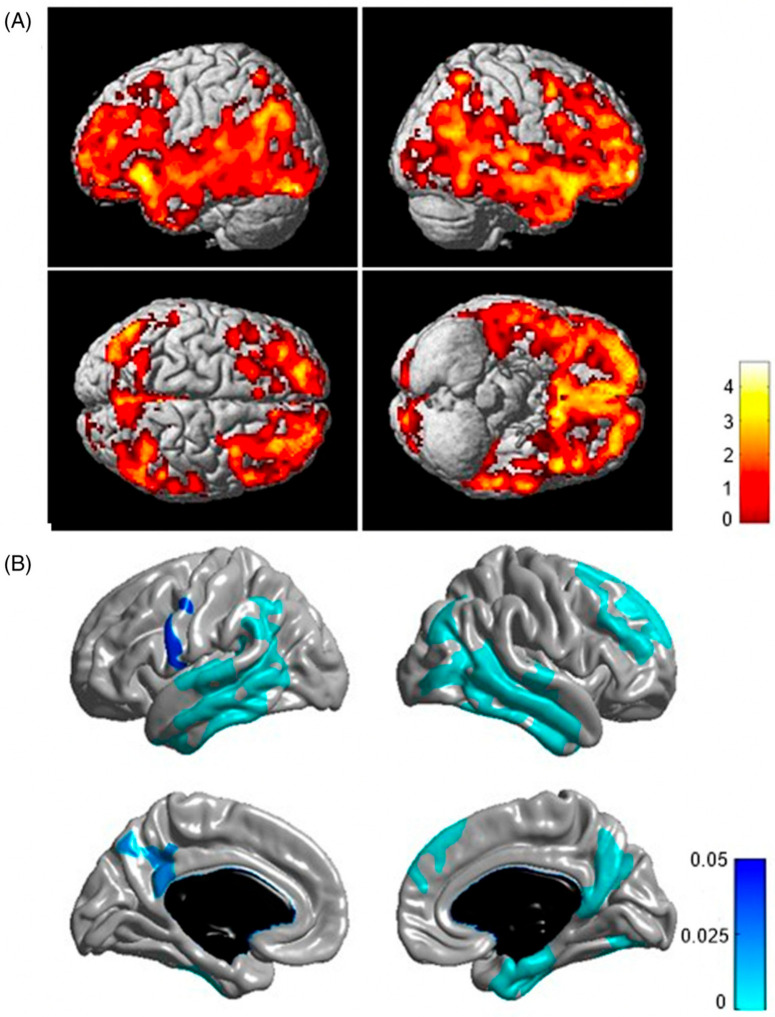
A whole-brain analysis conducted in the ADNI cohort assessed the association between rs10751647 and both (**A**) amyloid burden (via amyloid PET) and (**B**) cortical thickness (via MRI). Carriers of the minor allele showed reduced amyloid deposition, particularly in the frontal, parietal, and temporal lobes bilaterally, with statistical significance corrected using a false discovery rate threshold of 0.05. In addition, a greater number of minor alleles was linked to increased cortical thickness in the bilateral temporal regions, including the entorhinal cortex. These associations met significance criteria following random field theory correction at *p* = 0.05. Abbreviations: ADNI = Alzheimer’s Disease Neuroimaging Initiative; MRI = magnetic resonance imaging; and PET = positron emission tomography. Image reprinted without changes from Pyun et al. [41] under the Creative Commons Attribution 4.0 International License (CC BY). http://creativecommons.org/licenses/by/4.0/ (accessed on 12 June 2025).

In a complementary imaging genetics analysis in the ADNI cohort, Pyun et al. investigated rs10751647 and found that the minor alleles were associated with reduced amyloid PET SUVR and greater cortical thickness in AD-vulnerable regions, including the entorhinal cortex and bilateral temporal lobes (Figure 5) [41]. As detailed earlier, Kim HR et al. validated the *FGL2* locus in an independent Korean cohort [38].

Investigations involving non-European participants have identified unique protective and risk alleles, reinforcing the importance of inclusive recruitment. That said, many of these signals have yet to be confirmed in independent, external cohorts. This highlights the value of ancestry-specific studies for revealing genetic factors relevant to amyloid imaging phenotypes.

A summary of all major Amyloid PET GWASs included in this review can be found in Table 1. 

**Table 1:** Summary of the important findings related to GWAS of amyloid deposition on PET in Alzheimer’s disease in various studies; table made using Microsoft Excel 2025.

Study	Primary Gene Name	Gene Functions	Associated Functional Imaging	Number of Patients	Demographics
Zhao et al. (2016) [26]	*ABCA7*	*ABCA7*: Lipid metabolism	[^18^F]Florbetapir	1500	Caucasian (ADNI 2)
Roostaei et al. (2017) [36]	*IAPP*, *SLCO1A2*	*IAPP*: Amylin peptide, modulates Aβ-cognition interaction; *SLCO1A2*: organic anion transporter.	[^18^F]Florbetapir	678	Caucasian (ADNI 1, ADNI GO)
Apostolova et al. (2018) [25]	*APOE*, *ABCA7*, *CR1*, *FERMT2*	*APOE*: Lipid transport, Aβ clearance; *ABCA7*: lipid metabolism, Aβ phagocytosis; *FERMT2*: cell adhesion, APP trafficking.	[^11^C]PiB and [^18^F]Florbetapir	977	Caucasian (ADNI 1, ADNI 2, and ADNI Grand Opportunity)
Squillario et al. (2020) [29]	*TOMM40*, *GRM7*	*TOMM40*: Mitochondrial protein transport; *GRM7*: glutamate receptor signaling.	[^11^C]PiB and [^18^F]Florbetapir	500	Caucasian (ADNI 1 and ADNI 2)
Yan et al. (2021) [28]	*APOE, ADCY8, EFR3A, RAP2B*	*APOE*: Lipid transport, Aβ clearance; *ADCY8*: cAMP signaling; *EFR3A*: phosphoinositide metabolism; *RAP2B*: intracellular signaling.	[^11^C]PiB PET	1000	European American (ADNI 1)
Kim HR et al. (2021) [38]	*FGL2*	*FGL2*: Regulates coagulation and immune response; downregulation linked to reduced amyloid plaque deposition.	[^18^F]florbetapir and [^18^F]flutemetamol	1474	Korean (KBASE-V)
Eissman et al. (2022) [39]	*GATA3*, *TAF3, CDH18*	*PITPNM3*: Lipid signaling; *AP2A2*: clathrin-mediated endocytosis; *GATA3*: transcription factor, astrocyte/immune regulation; *TAF3*: chromatin remodeling; *CDH18*: cell adhesion in limbic regions.	[^18^F]Florbetapir	5024	Caucasian (ADNI 2 and A4)
Ali et al. (2023) [27]	*APOE*, *ABCA7*, *CR1*, *FERMT2*	*APOE*: Lipid transport, Aβ clearance; *ABCA7*: lipid metabolism, Aβ phagocytosis; *CR1*: complement-mediated Aβ clearance, immune regulation; *FERMT2*: cell adhesion, APP trafficking.	[^11^C]PiB and [^18^F]Florbetapir	13,409	Multiethnic (Knight ADRC A4, DIAN, ADNI)
Kim BH et al. (2023) [42]	*LCMT1, SCRN2, OSBPL7*	*LCMT1*: Phosphatase regulation; *SCRN2*: vesicle trafficking; *OSBPL7*: lipid transport and signaling.	[^18^F]flutemetamol	759	Korean (K-ROAD)
Patel et al. (2025) [37]	*SCN7A, SH3GL1, MFSD12, PITPNM3, AP2A2*	*SCN7A*: Sodium channel, neuronal excitability; *SH3GL1*: endocytosis, APP processing; *MFSD12*: membrane transport; *PITPNM3*: lipid signaling; *AP2A2*: clathrin-mediated endocytosis.	[^18^F]Florbetapir	1888	Caucasian, European

## 14. Limitations

This systematic review has several important limitations that warrant consideration. Most of the included studies have modest to moderate sample sizes (ranging from 500 to 13,409 participants), which significantly limits the statistical power and generalizability of the findings. Another critical limitation is the substantial overlap in study populations across the included investigations, with 8 of 10 studies utilizing data from the Alzheimer’s Disease Neuroimaging Initiative (ADNI) consortium either exclusively or in combination with other cohorts. This overlap means that many of the reported associations may reflect findings from largely the same individuals rather than independent replications, potentially inflating the apparent consistency of genetic associations and limiting the true generalizability of results beyond the ADNI population. The predominant reliance on ADNI data introduces potential selection biases inherent to this specific cohort, including its recruitment strategies, inclusion criteria, and demographic characteristics, which may not be representative of the broader global population affected by Alzheimer’s disease.

PET-GWASs also face unique methodological challenges that compound the effects of limited sample sizes. Unlike traditional case-control GWASs that rely on binary diagnostic classifications, PET-GWASs require quantitative imaging phenotypes that are inherently more variable and potentially influenced by technical factors, such as scanner differences, acquisition protocols, and image processing methodologies [28,43,44]. The modest sample sizes in many included studies may be insufficient to detect small-to-moderate effect sizes of genetic variants, particularly for non-*APOE* loci where effect sizes are typically smaller (β < 0.1). This limitation is particularly critical when considering that the integration of neuroimaging with genomics requires substantially larger sample sizes than traditional phenotype-based GWASs to achieve adequate statistical power for discovery and replication of genetic associations.

Furthermore, the heterogeneity in PET tracers ([^11^C]PiB (synthesized in-house at the University of Pittsburgh, Pittsburgh, PA, USA); [^18^F]Florbetapir (Amyvid), Avid Radiopharmaceuticals (Eli Lilly), Philadelphia, PA, USA; and [^18^F]Flutemetamol (Vizamyl), manufactured for GE Healthcare by Medi-Physics, Inc., Arlington Heights, IL, USA; imaging protocols, and quantification methods across studies introduces additional variability that may obscure true genetic associations. The combination of overlapping populations and methodological heterogeneity makes it difficult to distinguish between truly replicated findings and spurious associations that may arise from the same underlying dataset analyzed with different approaches. The modest sample sizes limit the ability to conduct robust subgroup analyses by tracer type, ancestry, or disease stage, potentially masking important effect modifications. These constraints are particularly relevant for loci with sex-specific effects or resilience associations, which require large, ancestrally diverse cohorts to detect and validate robustly. In the absence of this broader evidence, their use in clinical stratification or the design of sex-targeted therapeutic approaches cannot be reliably supported. Additionally, the predominance of studies in populations of European ancestry (8 of 10 studies) with only two studies in Korean populations severely limits the generalizability of findings to global populations, particularly given the demonstrated ancestry-specific effects observed for variants such as *FGL2*.

The lack of standardized outcome measures and the variability in covariate adjustment strategies across studies further complicate the interpretation of results. The heavy reliance on ADNI data also means that the field’s understanding of PET-GWAS associations may be disproportionately influenced by the specific characteristics, demographics, and potential biases inherent to this single consortium, rather than reflecting the true diversity of genetic influences on amyloid pathology across different populations and study designs. The absence of a formal risk of bias assessment and the fact that no protocol was registered prior to conducting this review represent additional methodological limitations. Finally, the rapidly evolving nature of both neuroimaging and genomic technologies means that some of the included studies may reflect earlier, less sophisticated analytical approaches that could influence the reliability and reproducibility of reported associations.

## 15. Conclusions

The use of amyloid PET in GWASs has significantly enhanced our knowledge of the genetic landscape associated with β-amyloid deposition in the brain. In addition to *APOE* ε4, which remains the most consistently replicated genetic risk factor, recent studies have identified several additional loci, including *ABCA7*, *FERMT2*, *CR1*, and *FGL2*, that may influence amyloid accumulation through mechanisms involving lipid metabolism, mitochondrial function, endocytic trafficking, immune regulation, and synaptic signaling.

Using quantitative assessment measures of amyloid burden rather than binary diagnostic categories enhances the sensitivity of gene discovery efforts and provides a nuanced understanding of how specific biological pathways contribute to AD risk. The functional specificity of loci such as *ABCA7*, the context-dependent effects of genes like *FERMT2* and *IAPP*, and the identification of sex-specific resilience variants highlight the complexity of genetic influences on amyloid pathology. Furthermore, the identification of ancestry-specific associations, for example, *FGL2* in Korean populations, reinforces the need for more inclusive and diverse cohorts in future studies.

An expanding body of research supports a polygenic framework in which multiple genetic loci, acting through diverse biological pathways, influence an individual’s susceptibility to amyloid buildup in the brain. As Alzheimer’s research continues to shift toward precision-based prevention and treatment strategies, these findings provide valuable guidance for refining biomarker profiles, identifying therapeutic targets, and advancing insights into Alzheimer’s disease progression. Continued research, particularly through well-powered and diverse prospective studies, is essential to validate these findings and uncover additional genetic contributors to amyloid pathology.

## Supplementary Materials

The following supporting information can be downloaded at https://www.mdpi.com/article/10.3390/jimaging11080280/s1, Supplementary Table S1: PICO framework used for study selection; Scheme S1: PRISMA Abstract Checklist; Scheme S2: PRISMA 2020 Checklist.
